# Draft Whole-Genome Sequence of Leishmania (Viannia) braziliensis Presenting Leishmania RNA Virus 1, from Western Amazon, Brazil

**DOI:** 10.1128/MRA.00924-18

**Published:** 2018-09-06

**Authors:** Andonai Krauze, Raul Maia Falcão, Marlon Grégori Flores Custódio, Iasmin Ferreira Pimentel, Lilian Motta Cantanhêde, Gabriel Eduardo Melim Ferreira, Elisa Cupolillo, Ricardo de Godoi Mattos Ferreira

**Affiliations:** a Laboratório de Genética Humana da Universidade Federal de Rondônia–UNIR, Porto Velho, Brazil; b Universidade Federal de Pernambuco, Recife, Brazil; c Laboratório de Epidemiologia Genética, Fundação Oswaldo Cruz, Fiocruz Rondônia, Porto Velho, Brazil; d Instituto Nacional de Epidemiologia da Amazônia Ocidental–EpiAMO, Rondônia, Brazil; e Fundação Oswaldo Cruz, Instituto Oswaldo Cruz–IOC, Rio de Janeiro, Brazil; University of California, Riverside

## Abstract

Leishmania (Viannia) braziliensis is the main etiological agent of tegumentary leishmaniasis in the neotropics. Here, we report a draft genome sequence (31.2 Mb) of an L. braziliensis strain from the western Amazon region of Brazil. This genome sequence will complement those available for other Leishmania species and contribute to further studies focusing on this parasite and the neglected diseases associated with it.

## Abstract (precis)

Leishmania (Viannia) braziliensis is the main etiological agent of tegumentary leishmaniasis in the neotropics. Here, we report a draft genome sequence (31.2 Mb) of an L. braziliensis strain from the western Amazon region of Brazil.

## ANNOUNCEMENT

Leishmania braziliensis (Kinetoplastida: Trypanosomatidae) belongs to the subgenus Viannia, which comprises species found exclusively in the neotropics; it is the main agent of tegumentary leishmaniasis (TL) in this region and causes a broad range of clinical manifestations, ranging from single to multiple lesions in the skin and nasopharyngeal mucosa, as well as persistent metastatic disease. The clinical expression of TL caused by L. braziliensis is multifactorial, being influenced by host and parasitic characteristics, including its endosymbiosis with Leishmania RNA virus 1 (LRV1) (1). The hypothesis that LRV1 is an ancient virus that coevolved with Leishmania species (2) and is not transmitted from one Leishmania strain to another is well accepted. Genetic clusters observed for L. braziliensis strains correlate with the presence/absence of LRV1, as well as with the phylogeny of this endosymbiont (3), indicating differences in the genomes of L. braziliensis strains bearing LRV1.

Some studies have revealed a considerable intraspecies variability in L. braziliensis (4, 5), which could explain its ability to adapt to different ecological conditions. There are around 30 Leishmania genome assemblies available, 6 corresponding to Leishmania (Viannia) species and only 2 *for* L. braziliensis. These genomes were obtained from long-term cultures and maintained using *in vivo* and *in vitro* conditions, which were recently demonstrated to affect genomic characteristics of this organism (6).

Here, we report the genome of an L. braziliensis strain (IOC-L3564) isolated in 2014 from a cutaneous lesion from a patient infected in the western Amazon region of Brazil and maintained with few *in vitro* passages. This is the first reported genome sequence of an L. braziliensis strain presenting the endosymbiont LRV1. The strain was typed as L. braziliensis by the *hsp*70 PCR gene restriction fragment length polymorphism protocol and isoenzyme electrophoresis (7, 8) and was deposited in the Leishmania collection at the Oswaldo Cruz Institute. LRV1 was detected by reverse transcriptase PCR (8). Genomic DNA was extracted from an *in vitro* culture and purified using a PureLink DNA minikit prior to library preparation. The library was prepared with an Ion Xpress Plus fragment library kit. Genomic libraries were enriched using an Ion PGM template Hi-Q View OT2 kit and sequenced using the Ion Torrent platform.

Using the TMAP tool for the Ion Torrent platform (https://github.com/iontorrent/TMAP), we determined the obtained reads to have 98.39% identity to the reference genome L. braziliensis strain M2904 (release TriTrypDB-27) (9). Trimming under the parameters TRAILING:5 LEADING:7 SLIDINGWINDOW:3:15 MINLEN:150 was performed using Trimmomatic (10). *De novo* assembly of the trimmed reads was conducted with SPAdes version 3.1.10 (11), which generated 10,557 scaffolds. The redundant scaffolds were removed using Redundans version 0.13a (12), which resulted in 7,363 scaffolds that were oriented and ordered on ABACAS version 1.3.1 (13) with default parameters; 34 scaffolds/chromosomes were obtained as the consensus for the haploid Leishmania genome (90.57% coverage), while 993 scaffolds did not match the reference genome L. braziliensis M2904 (release TriTrypDB-27).

QUAST software was used to check the quality of the assembly and to determine genome fraction (%) metrics (14). Annotation and gene prediction were performed on the Companion Server version 1.0.2 pipeline (15). Characteristics of the draft genome sequence of L. braziliensis IOC-L3564 are presented in Table 1.

**TABLE 1:** Characteristics of the draft genome sequence of L. braziliensis strain IOC-L3564, a parasite from western Amazon, Brazil, presenting LRV1

Parameter	Results
SRA no.	SRP144244
ENA assembly no.	GCA_003304975
GenBank accession no.	QFBG00000000
Total no. of reads	3,400,000
Read length (bp)	184
Coverage (×)	17.4
Draft genome size (bp)	38,003,648
GC content (%)	57.38
No. of contigs	8,090
*N*_50_ contig size (bp)	7,920
No. of scaffolds	1,029
*N*_50_ scaffold size (bp)	758,103
Total no. of genes annotated	8,278
No. of full-length genes	8,024
No. of partial-length genes	254
No. of genes with known function	4,023
No. of tRNAs	65
No. of snRNAs	2
No. of snoRNAs	5
No. of rRNAs	10
No. of pseudogenes	935

### Data availability.

This draft genome sequence has been deposited at DDBJ/ENA/GenBank under the accession number cited in Table 1.
